# Supplementary material for: Immune-Related Genomic Schizophrenic Subtyping Identified in DLPFC Transcriptome
Source: Genes (Basel). 2022 Jul 4;13(7):1200. doi: 10.3390/genes13071200 (PMC9319783; doi:10.3390/genes13071200)
Supplement: Supplementary file 1 [file genes-13-01200-s001.zip › Supplementary materials.pdf]

Supplementary Materials

Table S2. Illumina versus PolyA schizophrenic patient clustering. Number of patients classified as Type 1 or Type 2 for each dataset.

		PolyA	
		Type 1	Type 2
Illumina	Type 1	46	11
	Type 2	8	42

Table S3. PolyA versus RiboZ schizophrenic patient clustering. Number of patients classified as Type 1 or Type 2 for each dataset.

		RiboZ	
		Type 1	Type 2
PolyA	Type 1	57	5
	Type 2	24	30

Table S4. RiboZ versus Illumina schizophrenic patient clustering. Number of patients classified as Type 1 or Type 2 for each dataset.

		Illumina	
		Type 1	Type 2
RiboZ	Type 1	51	25
	Type 2	3	28

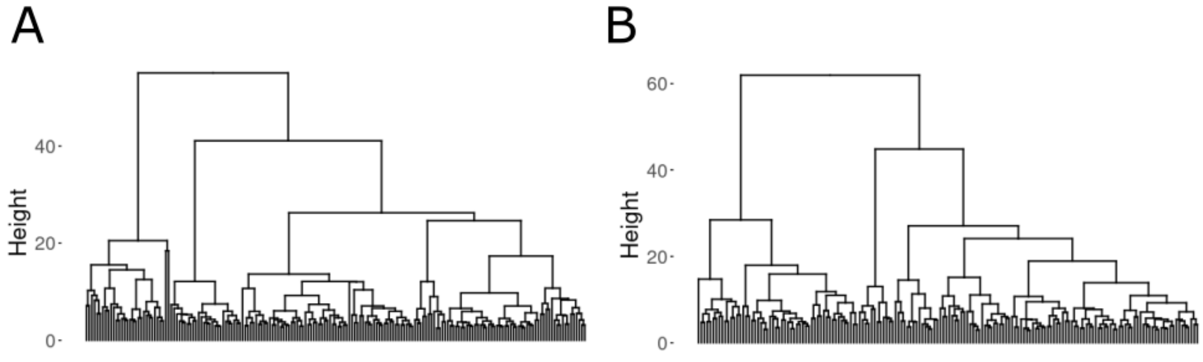

Figure S1. Unsupervised clustering of Controls (A) and schizophrenics (polyA) (B). Controls were analyzed in the same pipeline as schizophrenics (see *Methods and Materials* for further details). Unsupervised hierarchical clustering does not show distinct grouping in controls, unlike in PolyA schizophrenics which has two main groups separated by a larger distance than controls.

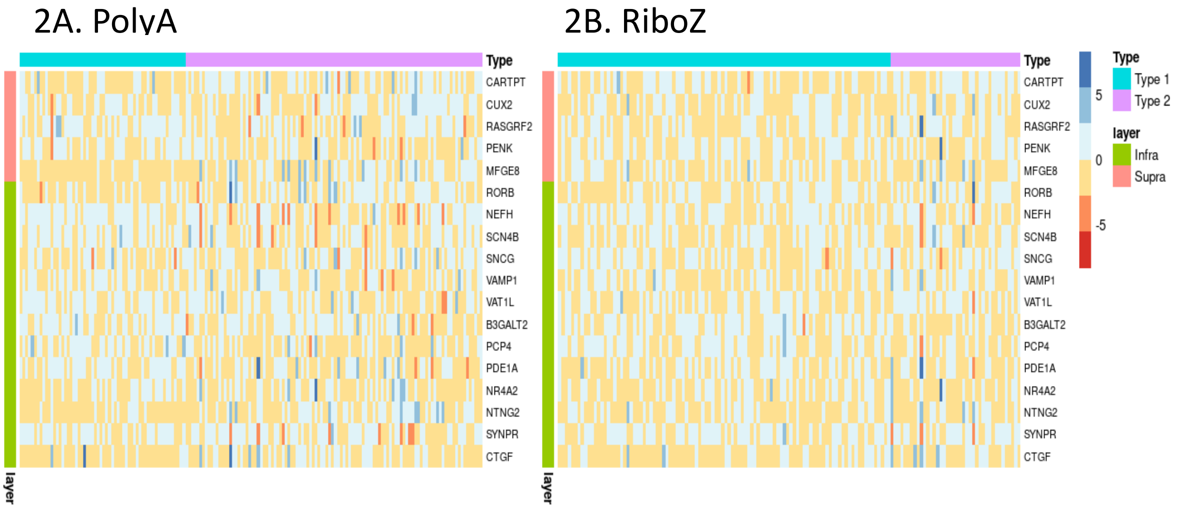

Figure S2. Gene expression of supra- and infra- layer associated markers in polyA (A) and riboZ (B). In both datasets, there doesn't appear to be any large differences in expression of sugra- or infra-granular genes between Type 1 and Type 2.

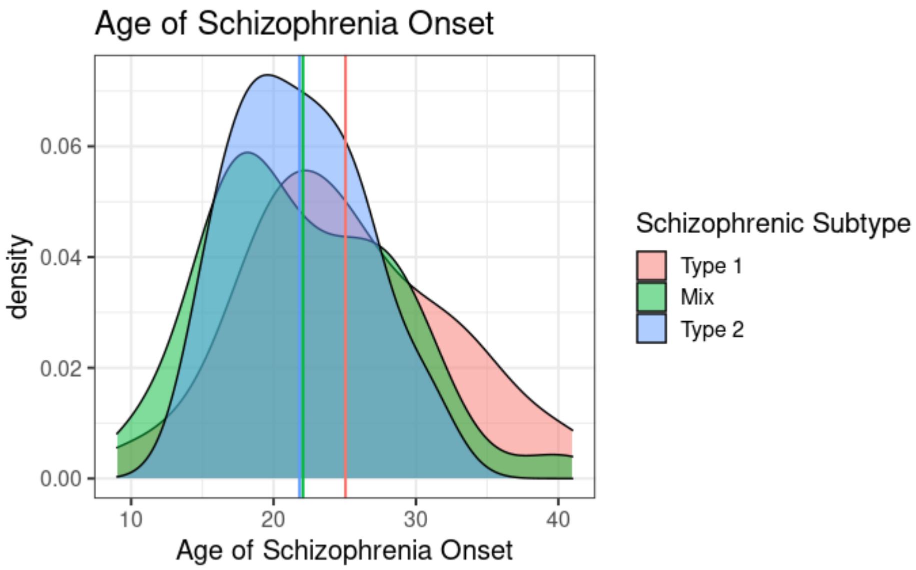

Figure S3. Age of schizophrenia onset distribution for each SCZ subtype. We can see that the age of schizophrenia onset does not vary significantly between type 1 (pink), mix (green) or type 2s (blue). Colored lines indicate average age of schizophrenic onset for that group. Tukey HSD, p value > 0.05.

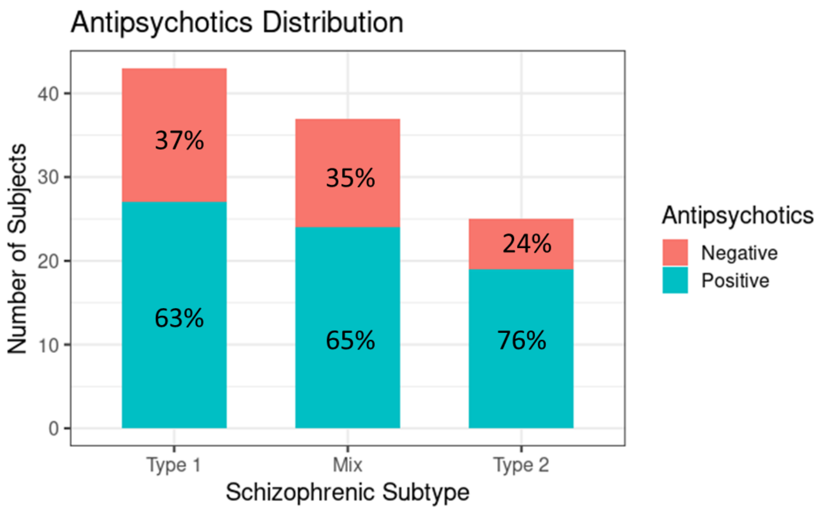

Figure S4. Antipsychotic distribution for each SCZ subtype. The ratio of subjects positive (blue) and negative (pink) for antipsychotic drugs does not vary significantly for Type 1s, Mix and Type 2s. Tukey HSD, *p*-value > 0.05.

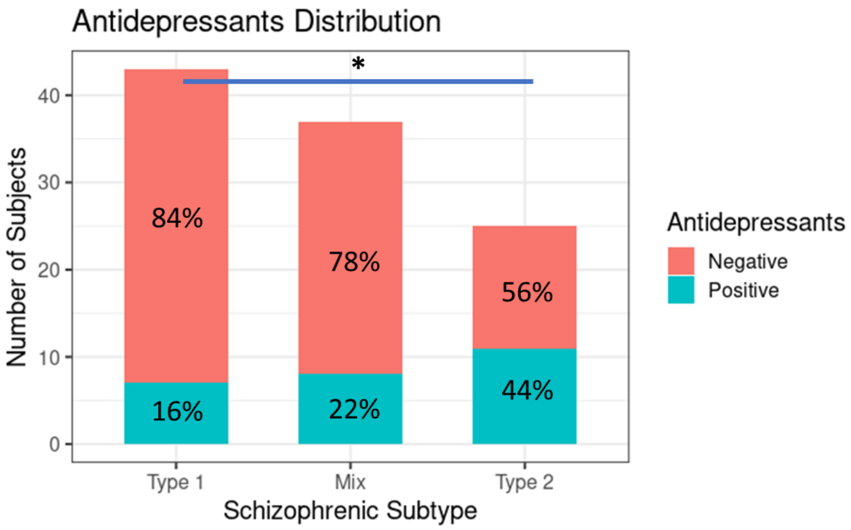

Figure S5. Antidepressants distribution for each SCZ subtype. We observe that there is a statistically significant difference in the ratio of subjects positive (blue) and negative (pink) for antidepressant drugs between Type 1 and Type 2s only. However, we see that for all three subtypes a majority of subjects are negative for antidepressants is unlikely to be driving the clustering. Tukey HSD, * *p* value < 0.05.

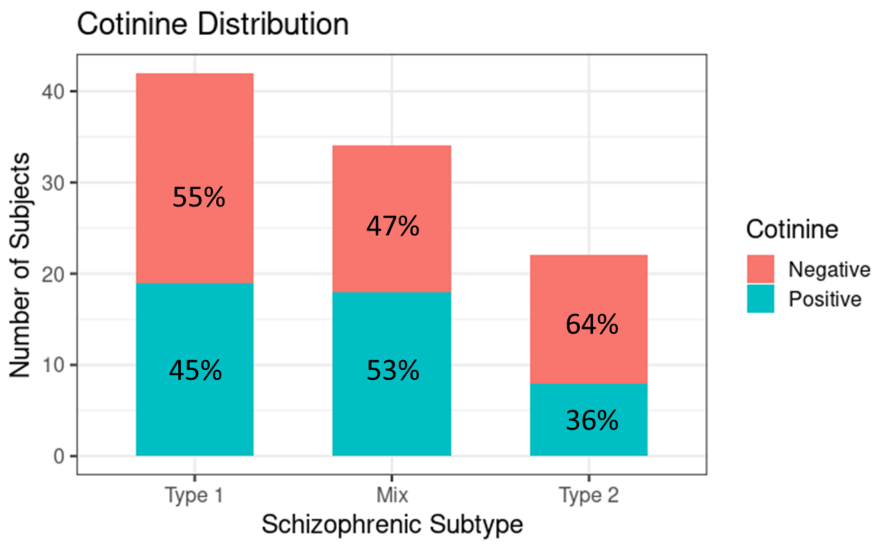

Figure S6. Cotinine Distribution for each SCZ subtype. Schizophrenia and smoking are strongly associated; however, this association does not seem to be responsible for the Type 1, Mix and Type 2 subtypes. Tukey HSD, *p* value > 0.05.

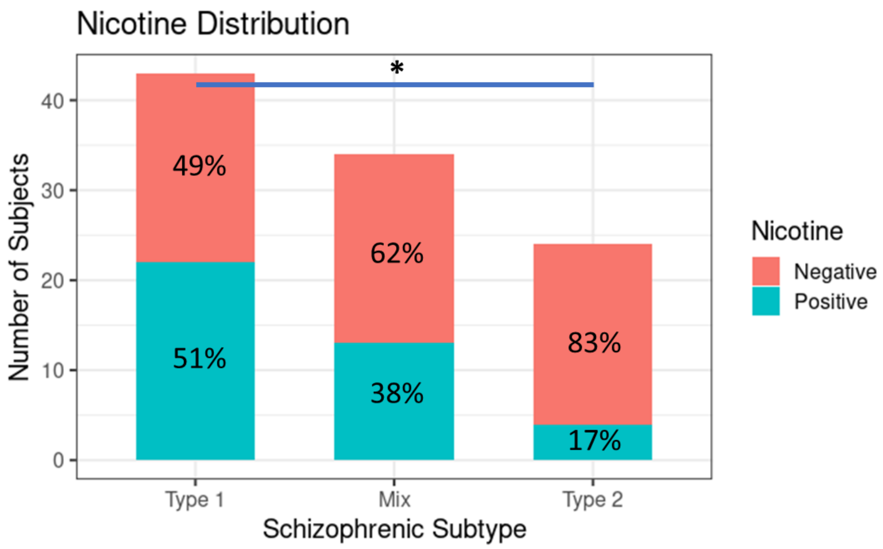

Figure S7. Nicotine distribution for each SCZ subtype. We observe that there is a statistically significant difference in the ratio of subjects positive (blue) and negative (pink) for nicotine between Type 1 and Type 2s only. However, we see that for all three subtypes most subjects are negative for nicotine and is unlikely to be driving the genetic clustering.

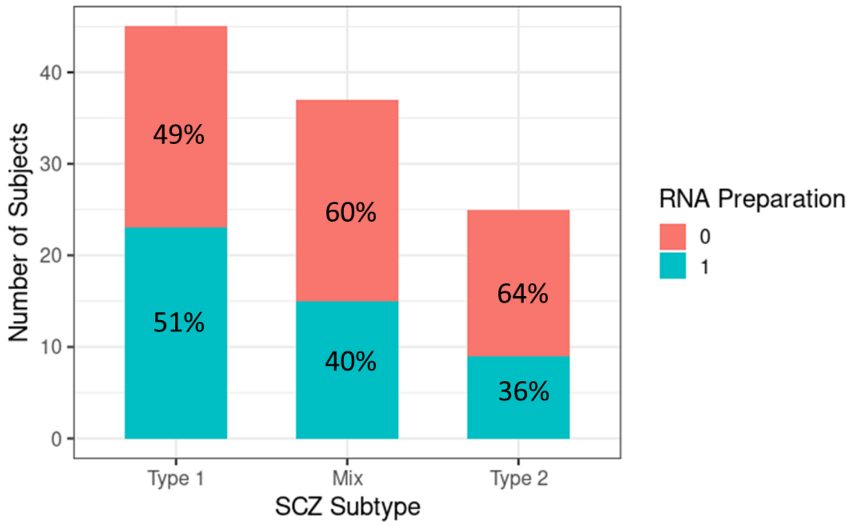

Figure S8. RNA Preparation for each SCZ subtype. Between polyA and riboZ, not all samples came from the same RNA preparation. However, whether the RNA preparations were the same (1, blue) or different (0, pink) did not significantly influence the SCZ subtypes. Tukey HSD, p value > 0.05.

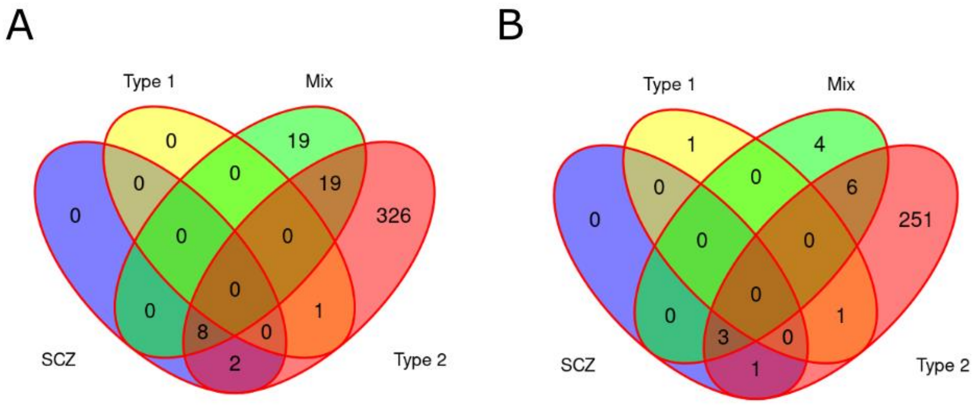

Figure S9. Venn diagram of overlapping genes with logFC greater than 1 across the SCZ groups. Number of genes that are shared between groups will be listed in intersecting circles. Type 2 genes have over 200 genes that have a logFC greater than 1, and a majority of these genes are not shared with the other SCZ groups.
